# Supplementary material for: Robust two-stage influenza prediction model considering regular and irregular trends
Source: PLoS One. 2020 May 21;15(5):e0233126. doi: 10.1371/journal.pone.0233126 (PMC7241782; doi:10.1371/journal.pone.0233126)
Supplement: S1 Table — (DOCX) [file pone.0233126.s001.docx]

| influenza type a  bronchitis  spring break family  treating the flu  type a influenza | duration of flu  cough fever  flu medicine  cold or flu  contagious flu | symptoms of the flu  reduce fever  having the flu  early flu symptoms  flu remedies | getting over the flu  signs of the flu  length of flu  fever flu  dangerous fever |
| --- | --- | --- | --- |
