# Supplementary material for: Robust two-stage influenza prediction model considering regular and irregular trends
Source: PLoS One. 2020 May 21;15(5):e0233126. doi: 10.1371/journal.pone.0233126 (PMC7241782; doi:10.1371/journal.pone.0233126)
Supplement: S2 Table — (DOCX) [file pone.0233126.s002.docx]

| インフル  (flu)  インフルエンザ  (influenza)  頭痛  (headache)  タミフル  (tamiflu)  風邪  (cole) | A型  (Type A)  B型  (Type B)  体調  (physical condition)  熱  (fever)  微熱  (slight fever) | 流行  (epidemic)  いんふるえんざ  (influenza in hiragana)  潜在期間  (potential period)  予防  (prevention)  薬  (medicine) | ワクチン  (vaccine)  ウイルス  (virus)  予防接種  (vaccination)  咳  (cough)  マスク  (mask) |
| --- | --- | --- | --- |
