# Supplementary material for: Robust two-stage influenza prediction model considering regular and irregular trends
Source: PLoS One. 2020 May 21;15(5):e0233126. doi: 10.1371/journal.pone.0233126 (PMC7241782; doi:10.1371/journal.pone.0233126)
Supplement: Attachment — Submitted filename: rebuttal_letter_plos_one2020.pdf [file pone.0233126.s003.pdf]

Dear Dr Tzai-Hung Wen
Academic Editor, PLOS ONE

Thank you for inviting us to submit a revised draft of our manuscript (PONE-D-19-34966) entitled "Robust Two-stage Influenza Prediction Model considering Regular and Irregular Trends" to the PLOS ONE. We also appreciate the time and effort you and each of the reviews have dedicated in providing insightful feedback on ways to strengthen our manuscript. Thus, it is with great pleasure that we want to re-submit our manuscript for further consideration. We have incorporated changes that reflect the detailed suggestions you have graciously provided. We also hope that the edits and the responses we have provided satisfactorily address all the issues and concerns you and the reviewers have noted. In this rebuttal letter, the comments or suggestions from you and the reviewers are inside the black frames, our responses follow them and our revisions in the manuscript are shown in italic.

Best regards,
Taichi Murayama

To the editor:

> Please ensure that your manuscript meets PLOS ONE's style requirements, including those for file naming.

Thank you for your comment. We have already checked that our manuscript meets your style requirements. Please feel free to let us know if something else needs to be revised.

> In your Methods section, please include additional information about your dataset and ensure that you have included a statement specifying whether the collection method complied with the terms and conditions for the website.

Thank you for your comment. Our collection method complied with the terms and conditions for these sites (Google Trend and ILI data collection sites). We have described this in the Material and Method Section.

P4 Lines 98-100
Like the US data, we normalized GT data such that the time series of GT data had a zero mean and a standard deviation of one.
Our collection method complied with the terms and conditions for these data provision sites.

> We note that one or more of the authors is affiliated with the funding organization, indicating the funder may have had some role in the design, data collection, analysis or preparation of your manuscript for publication; in other words, the funder played an indirect role through the participation of the co-authors. If the funding organization did not play a role in the study design, data collection and analysis, decision to publish, or preparation of the manuscript and only provided financial support in the form of authors' salaries and/or research materials, please do the following:
> …

Thank you for your comment. The funding organization only provided financial support in the form of authors' salaries or research materials. We agree with the following statement. Could you please change the online submission form as

follows?

“The funder provided support in the form of salaries for Dr Nobuyuki Shimizu and Mr Sumio Fujita, but did not have any additional role to pay in the study design, data collection, analysis, decision to publish, or preparation of the manuscript. The specific roles of these authors are articulated in the ‘author contributions’ section.”

> Please provide an amended Competing Interests Statement that explicitly states this commercial funder, along with any other relevant declarations relating to employment, consultancy, patents, products in development, marketed products, etc.
> Within this Competing Interests Statement, please confirm that this does not alter your adherence to all PLOS ONE policies on sharing data and materials by including the following statement: "This does not alter our adherence to PLOS ONE policies on sharing data and materials.” (as detailed online in our guide for authors http://journals.plos.org/plosone/s/competing-interests). If there are restrictions on sharing of data and/or materials, please state these. Please note that we cannot proceed with consideration of your article until this information has been declared.
> Please include your amended Competing Interests Statement within your cover letter. We will change the online submission form on your behalf.

Thank you for your comment. We confirmed that our adherence to PLOS ONE policies does not change and sharing of data and/or materials has no restrictions. We added the following sentence: “The authors have declared that funding from a commercial source, Yahoo Japan Corporation, did not alter our adherence to PLOS ONE policies on sharing data and materials.” in the Competing Interests Statement. Could you please change our Competing Interests Statement as above?

To Reviewer #1:

Page 5, Lines 131. Is there any reason to support the selection of AR(26)?

Thank you for your comment. Our study proposes the architecture of combining historical ILI data and UGC data for predicting flu volume. The AR model can be replaced with another model, as described in P10 Lines 285-286. This paper applied the AR model as the simplest model to learn seasonality from past time series.

The reason of selecting AR(26), which uses 26 weeks (a half year) of ILI data, was to consider seasonal trends. We expected to capture sufficient seasonality by inputting data for half a year. To consider seasonal trends, AR(52) is the best to capture one-year trend. However, the number of test verifications decreases because more data are required for training when we use AR(52) as the 1st model. It is important to test an epidemic prediction model like ours for more than one year to verify its effectiveness because the data of a time series in an epidemic disease significantly change depending on the year.
We chose AR(26) for capturing seasonality and for performing more test runs than when using AR(52) as the 1st model.
We have modified the explanation of selecting AR(26) more in detail as follows:

P5 Lines 137-143
Our paper uses the AR model as the 1st model. The input data for the AR model is the total weekly ILI rate/number data. It is aggregated by medical institutions in each area. We selected AR(26), which uses 26 weeks (a half year) of ILI data as predictors, and attempted to capture the seasonality of half a year. Although AR(52) would be the best to capture year-long seasonality as a year accounts for 52 weeks, it practically requires more data in training. Thus, it is difficult to perform a sufficient number of test verifications.

Figure 4. When comparing the difference values of each model, ARGO demonstrated a different direction of the deviance comparing to two-stage model and Random Forest approach. Is there any interpretation for the phenomena?

Thank you for your comment. In accordance with the reviewer's comment, Fig 4 shows that ARGO tended to deviate upward, while the two-stage model and random

forest tended to deviate downward. To interpret the phenomena, we need observation periods longer than two-years for the flu prediction experiments. We cannot judge only on the basis of our experiments whether the phenomena were accidental or not. We added the following explanations of the tendency and the future issues to the Model Comparison Section.

P9 Lines 262-266

The obs. vs. predicted scatter plots illustrate the prediction result of each model by period and country. It shows that ARGO tended to deviate upward while the Two-stage model and random forest tended to deviate downward. We need a longer observation period for the interpretation of the phenomena. The verification of how each model works for flu prediction is an issue for future studies.

The result and discussion mainly focus on the performance comparison of different models. The content of discussion seems missed in this part. It' s better to add more detail discussion with previous literature in the manuscript.

Thank you for your comment. We agree that it is better to add more detailed discussions regarding the previous literature. Accordingly, we have added some discussions about the role of our paper compared to previous literature in the "Strengths and Weaknesses of the Two-stage model in Discussion" Section as follows.

P9 Lines 278-299

Strengths and Weaknesses of the Two-stage model

Throughout the experiments, we demonstrate that historical ILI data and UGC data are useful for flu prediction, which is consistent with the results of previous studies [7,12,13]. One salient feature of our model is that the time series data spans are divided clearly into regular trend parts and irregular trend parts. We show that the proposed model, which processes the historical data separately from the UGC data as input, achieves better performance than the existing models that use these two input data simultaneously. Although this study employed the AR model as the 1st model, we could replace this part with any other models such as RNN[29] and disease model[21].

The proposed method has an advantage not only in performance, but also in robustness against outlier data. Noise and outliers included in UGC data, which are sometimes caused by transaction delays under heavy traffic or malfunctions in crawling, always hinder epidemic prediction tasks. It is crucial to build a model which is robust against outlier data for prediction, but most researches, except [22] and ours, have ignored the issue.

However, the proposed model has some limitations. Our approach depends strongly

on the historical model produced by the 1st model. In flu prediction, the 1st model itself performs very well, which is suitable for our approach. However, in other targets such as infectious gastroenteritis (mainly on norovirus) and Zika fever, which do not show any seasonal trends, the model mostly relying only on UGC data might perform better [13]. In future studies, a large scale comparative investigation that covers more infectious diseases would be desired.

To Reviewer #2:

My major concern is about the methods. Methods section lacks enough description of the main procedures of the Two-stage model. Very few words were used to describe the model. Author said "We designate the combination of the 1st Model and the 2nd Model as the Two-stage Model", but how were these 2 models combined isn't described. More details are needed to illustrate how to combine the two model's results to obtain the final prediction value. Does the proposed approach only add the two models' results? Or with any weights?

Thank you for your comment. We apologize because the description of the two-stage model is lacking. The Two-stage model outputs the prediction results by only adding the results of two models (without any weights), as shown in Fig 3. Each model is trained to predict a regular trend value and a deviated value from this; thus, we need no more weights to adjust them.
We added more details of the Two-stage model in "Basic Idea" in the "Method: Two-stage model" Section as follows:

P4 Lines 103-124
Our methodology can accommodate data of two types: historical data and UGC data, which have different benefits and shortcomings. To exploit the benefits of each kind, we proposed a novel method that divides the prediction process into two stages – the 1st model and the 2nd model – where each model uses different types of data.

1 1st model for regular trends: First, an autoregressive model is used to learn and predict the regular movements from only the historical data. We designate this model as the 1st model, which makes the basis of the succeeding model. The model predicts the future ILI rate/patient number from the historical data.

2 2nd model for irregular trends: This model is designed to predict sudden outbreaks. To identify sudden outbreaks, the model learns and predicts the difference between the ground truth and the predicted values by the 1st model using GTs data, in a different training term from the 1st model training term.

We designated the combination of the 1st model and 2nd model as the Two-stage model. Thus, the final prediction values of the Two-stage Model are calculated as the sum of the output value of the 1st model and that of the 2nd model. As each model is trained to predict a regular trend value and a deviated value from this, no coefficient weights are required to adjust them.
Figure 2 shows the example of the prediction by the Two-stage model: the 1st model first predicts future ILI rate/patient number as regular trends from historical data (blue point), and then, the 2nd model predicts the differences between the output of the 1st model and the actual value (red dotted arrow), as irregular trends. The Two-stage model outputs the sum of the output of the 1st and 2nd model.

Also, how model 2 works with model 1' s outcome is not described. Eq(2) is not enough to understand how ILI rate from model 1 outcome can be used for model 2. Although "xt and ot-1 denote a current input and previous output vectors…" is described, what actual data are x and o, and how these variables correspond to ILI rate results from model 1.

Thank you for your comment. As mentioned earlier, the output of the Two-stage model is the sum of the two model outputs. The 2nd model is intended to predict the difference between the output of the 1st model and the observed target value such as the ratio/number of patients; it does not use the output of the 1st model as input data. Consequently, the variable $\boldsymbol{o_t}$ denotes the difference between the output of the 1st model and observed values. We added this point to the description of Eq(2).

P5 Lines 155-161
Our paper uses the LSTM as the 2nd model. The input data for LSTM are the weekly search query frequency data obtained through GT. We use 26 weeks of GT data before each prediction point as predictors. We predicted the differences between the ground truth and the predicted values by the 1st model. In Equation (2), $\boldsymbol{o_t}$ *denotes the predicted difference at time* $\boldsymbol{t}$. *In Figure 2, we show the usage of LSTM mentioned above for our experiment; such usage is very similar to the method used in a previous study [30].*

The introduction section should look like a concise summary of the research topic. I suggest the authors could show some previous studies' outstanding achievements based on the hospital surveillance data and UGC data (e.g. the quantity comparison of forecasting results based on the historical data and UGC data) rather than only explain the datasets.

On Page2, Line 19, cite some particle filtering approaches done by Yang W and Shaman J for influenza forecasting.

Thank you for your comment. We add the articles by Yang W and Shaman J and have shown the outstanding achievements of previous studies in the Introduction. We have added more descriptions of previous studies on P.2 Lines 14-62.

P2 Lines 14-62

Historical data: Historical time series data, called historical data, have a feature that potentially involves crucially important information such as marked seasonality. Therefore, approaches based on historical data have been reported [7, 8], and various models [12,19,22] have been proposed. Kane et al. [18] compared the effectiveness of flu prediction of an autoregressive integrated moving average (ARIMA) model and a regression model based on random forest. Nasserie, T., et al. [20] used disease models such as the IDEA model to project influenza peaks and epidemic final sizes. Some researches [29-31] used long short-term memory (LSTM), a neural network model, to verify its effectiveness in flu prediction. Yang, Wan., et al. [34] showed that the application of statistical filtering methods to epidemiological models makes reliable influenza prediction possible by comparing the performance of six state-of-the-art filter methods such as particle filters. These researches improved the prediction performance and showed the effectiveness of data intensive prediction techniques of flu epidemics. Although numerous efforts have been made, these approaches suffer from one limitation; they are insufficient to discriminate against various unexpected sudden movements.

UGC data: Another line of flu prediction research typically utilizes UGC text data or search query logs to seek signals of epidemic-related activities from crowds of users. To capture irregular movements detected by these signals, most studies have employed UGC on the Web [25] including search queries [4, 5, 7, 12, 13], microblogs [3, 13, 14], and access logs to Web pages such as Wikipedia [4] for flu prediction. Although a comparison of these resources has been presented in earlier reports [4, 10, 11], it still requires active discussions regarding which resources are the most useful for predicting flu epidemics. Signorini, A., et al. [11] examined the volume in posts including keywords related to influenza in the Twitter stream and showed the usefulness of twitter data for tracking flu epidemics. In addition, some have studies tackled the aspects such as which keywords on Google are useful [16] and when and where the model based on Twitter works well [14]. Various posts and search queries by users in UGC are important as signals to identify the beginning of epidemics and are used for the prediction of various epidemics [33] apart from influenza. Currently, Google Flu Trends (GFT) [5] is one of the most representative systems using UGC, which was designed to estimate the current ILI rate using Google search terms related to ILI. A benefit of using UGC data is that they can quickly detect the signals from online activities of a crowd of users. Nevertheless, UGC-based ILI prediction has some limitations: UGC exhibits difficulty in capturing crucially important regular trends such as long term annual movements in the time series of historical flu data, which might help the prediction of flu outbreaks [9].

In conclusion, these materials have their own advantages and disadvantages. They mostly correspond to two phenomena of epidemic management: (1) interpreting the intrinsic time series (regular trend) and (2) interpreting a sudden unexpected epidemic (irregular trend). Several studies [7, 13, 28] described the model of predicting the influenza volume using historical data and UGC data simultaneously and showed the effectiveness of the combination of each resource simultaneously. Instead of simultaneously, this study separately employs the two resources; the historical data are applied for regular trends, and the UGC data, for irregular trends (Figure 1). Our approach is practically useful because we can train the models separately; in case a UGC provider changes its service, our model would only have to train the UGC model. Our model is also robust against noise and outliers in data because the model is divided into two parts: regular prediction using historical data and irregular trend prediction using UGC data. We validated our hypothesis regarding the robustness of the proposed method throughout the experiment in "Robustness" of the Results Section.

[32] Shaman, Jeffrey, et al. "Real-time influenza forecasts during the 2012-2013 season." Nature communications 4.1 (2013): 1-10.
[33] Masri, Shahir, et al. "Use of Twitter data to improve Zika virus surveillance in the United States during the 2016 epidemic." BMC public health 19.1 (2019): 761.
[34] Yang, Wan, Alicia Karspeck, and Jeffrey Shaman. "Comparison of filtering methods for the modeling and retrospective forecasting of influenza epidemics." PLoS computational biology 10.4 (2014).

On page 2, Ln 42, “We also think that our model is strong against noise and outliers in data”. Please change the work “think” to other words and describe your analytical results to support your statement. People think is not enough to support this conclusion.

Thank you for your comment. In accordance with the comment, we have changed this text as follows:

P2 Lines 59-62

Our model is also robust against noise and outliers in data because the model is divided into two parts: regular prediction using historical data and irregular trend prediction using UGC data. We validated our hypothesis regarding the robustness of the proposed method throughout the experiment in "Robustness" of the Results Section.

What are the search queries used? Letting readers know these, e.g., by

including it in supplementary materials, would be good.

Thank you for your comment. We added search queries used for the model in the Supporting information. Table S1 shows the queries in English, and Table S2 shows the queries in Japanese.

P11 Lines 312-314

Supporting information

Table S1 Queries in English.

Table S2 Queries in Japanese.

Additionally, the merits of choosing ILI rate for US data and ILI patients for JPN data is not very clear to me.

Thank you for your comment. It is true that many studies on flu prediction used the ILI rate as the main target. The reasons for selecting ILI patients in Japan data are as follows: the data are provided in the format of the number of ILI patients, and the number of ILI patients is generally used rather than the ILI rate in Japan.

These aspects are described in P3 Lines 76-77 "NIID reports data on the number of patients with ILI symptoms every week in Japan" and P3 Lines 80-81 "the ILI patient number is more generic than the ILI rate in Japan, unlike in the US".

Some variables in eq(2) are not expounded and need to be so.

Thank you for your comment. We have added the description of variables in Eq(2) as follows:

P5 Lines 147-154

An LSTM cell works as a memory to manage information according to the decisions specified by the input $\boldsymbol{I}$*, output* $\boldsymbol{Y}$*, and forget gates* $\boldsymbol{F}$*. Each memory cell is implemented as shown below.*

Here, W_x *and* W_m *are adaptive weights and* $\boldsymbol{b}$ *is the intercept, initialized randomly in the range (0, 1). In addition,* x_t *and* o_{t-1} *denote the current input and previous output vectors, respectively. The current cell state is denoted as* C_t*;* σ *denotes the sigmoid function; and* $\odot$ *denotes the Hadamard product.*

The mechanism shown as Figure 2 should be described in more details. I assume the solid black line represent the previous historical data. Then author should describe all the remaining dots (2 blue dots, one black, one red) following the order of the steps. For example, does left blue dot (prediction

from the 1st model) occur before the above black dot. How is the 2nd blue dot predicted? And how is the red dot predicted? Why there is not red dot above the 1st left blue dot? Please describe the scenario more.

Thank you for your comment. We will describe the reason why there is no red dot above the 1st blue dot. The red dot is produced by combining the prediction of the 1st model from historical data prior to the time point of the 2nd blue dot and the prediction of the 2nd model from GT data more than one week prior to the time point of the red dotted arrow. To calculate the 2nd blue dot, we also need historical data of 1 week earlier, which are not provided by the influenza surveillance reporters, in addition to the historical data more than two weeks prior to the time point of the 2nd blue dot. Therefore, we first predict the 1 week preceding result of the 1st model indicated by the 1st blue dot and then predict the 1st model from the 1st blue dot and historical data; the result of the latter prediction is indicated by the 2nd blue dot. (described in P. 3 L.78-79 and P. 6 L.176-178). The same situation, where there is no red dot above the 1st blue dot, is illustrated in Figure 3.
For example, the 1st Model outputs regular trends in the 50th and 51st week because of the characteristic of the AR model, using the ILI report preceding the 49th week when we predict the 51st week flu volume. The 2nd Model outputs the gap in the 51st week using GT data prior to the 50th week.

We agree Figure 2 requires more detailed data. Thus, we added a more detailed description of the mechanism to the caption of Figure 2 and added a new sub-section, Two-stage model, in the Implementation Section.

Figure 2 Caption:
Outline of the Two-stage model: Black points in the upper graph indicate the observed values in each week. A Two-stage model produces a one-week ahead flu prediction by a combination of the 1st and 2nd models. The 1st model, the input of which is the time series of historical data, predicts future ILI rate/patient number as regular trends. The 1st model predicts the number of patients of this week and one-week ahead, given the historical data until the last week. The outputs of the 1st model are shown as blue points in the upper graph. Then, the 2nd model predicts irregular trends, which are differences between the predicted values and the actual values. The input for the 2nd model is time series data of the weekly total search frequencies of a set of predefined queries. The output of the 2nd model is shown as a dotted red arrow in the upper graph. The output of the Two-stage model is indicated by a red point, which is the simple sum of the outputs of the 1st and 2nd models.}

P4 Lines 126-129

An autoregressive model (AR model) [27] is used for the 1st model. A LSTM model [29-31] is employed for the 2nd model. The Two-stage model calculates the sum of the output value of the 1st model and that of the 2nd model without any coefficient weights, as follows:

P5 Lines 162-172(new added sub-chapter)

The Two-stage model

The Two-stage model outputs the sum of the output of the 1st model (AR model) and the 2nd model (LSTM). In our setting, the AR model first outputs the prediction of the ILI rate/patient number from historical data prior to more than two weeks, and then, the LSTM model outputs the prediction of sudden outbreaks, which is the difference between the ground truth and the output of the AR model. We predict the number of influenza patients at time $t+1$, as formulated as follows:

$$\widehat{y_t^{1st}} = AR(y_{t-1}, y_{t-2}, \cdots y_{t-26})$$
$$\widehat{y_{t+1}^{1st}} = AR(\widehat{y_t^{1st}}, y_{t-1}, \cdots y_{t-25})$$
$$\widehat{d_{t+1}^{2nd}} = LSTM(g_t, g_{t-1}, \cdots g_{t-25})$$
$$\widehat{y_{t+1}} = \widehat{y_{t+1}^{1st}} + \widehat{d_{t+1}^{2nd}}$$

The AR model, as the 1st model, predicts a regular trend, $\widehat{y_t^{1st}}$ from the 26 weeks of the historical data y. The LSTM model, as the 2nd model, predicts the irregular trend $\widehat{d_{t+1}^{2nd}}$ from 26 weeks of GT data g. The output of the Two-stage model, $\widehat{y_{t+1}}$ is calculated by adding $\widehat{y_{t+1}^{1st}}$ and $\widehat{d_{t+1}^{2nd}}$.

In Table 1, why the periods in training using Model1 and Model2 are different. Should both models use the same periods for training?

Thank you for your comment. We built each model to play different roles separately. The 1st model is trained for predicting the ILI rate/patient from the input of the time series of historical data. The 2nd model is trained for predicting the difference between the prediction of the trained 1st model and observed values. This means that the 2nd model is trained to predict the value, which is never trained by the 1st model, using UGC data.

When the 1st and 2nd models are trained in the same period, very few differences are observed because the 1st model has already been trained to fit the true value (the observed ILI rate/patients). We need to set the training period of the 1st model prior to that of the 2nd model for capturing the difference between the prediction of the trained 1st model and the observed values, as the target of the prediction by the 2nd model.

The values given in parentheses are the difference of accuracy scores between

the original data and processed data with outliers processed data. Please describe more details what is processed data and how did author process the data.

Thank you for your comment. In accordance with the comment, we added details of how we processed the data for the experiment.

P6 Lines 192-205

We also prepared datasets with outliers to evaluate the robustness of the proposed model. UGC data sometimes exhibit disadvantages against noise (outliers) due to malfunctions in crawling or those in services because of heavy traffic. These problems lead to confounded prediction and time-consuming preprocessing of the data. Robustness against noise and outliers is important. Thus, we investigated the robustness of the Two-stage model. ILI data is not processed because it is less susceptible to contain outliers. GT datasets with outliers were created by randomly changing the frequency values of GT data in each query at each time span to 0.0 (the minimum value) or to the maximum value in each dataset without assuming any statistical distribution. Based on the above processing, three datasets with different ratios of outliers; 5.0%, 10.0%, and 15.0%, were created from the US GT data and the Japanese GT data, respectively. As described in the experimental settings, we conducted the same experiments on the prediction of the ILI rate and the number of ILI patients in the US and Japan from the 40th week of 2017 to the 39th week of 2018.

on the sentence "The model presented herein might not work well in some countries": if the authors decide to include this sentence, they should preferably give clear reasons why and how the model can be made to work in such countries.

Thank you for your comment. Clearly stating the necessary condition where the method works, we modified the sentence as below:

P11 Lines 307-309

Our model is only applicable to countries or regions where the amount of historical data is sufficient, which is the resource of the 1st model.
